# Blood biomarkers of post-stroke depression after minor stroke at three months in males and females

**DOI:** 10.1186/s12888-022-03805-6

**Published:** 2022-03-03

**Authors:** Xiuli Qiu, He Wang, Yan Lan, Jinfeng Miao, Chensheng Pan, Wenzhe Sun, Guo Li, Yanyan Wang, Xin Zhao, Zhou Zhu, Suiqiang Zhu

**Affiliations:** 1 Department of Neurology, Tongji Hospital, Tongji Medical College, Huazhong University of Science and Technology, 1095 Jiefang Avenue, Wuhan, 430030 Hubei China; 2 Department of Medical Affair, Tongji Medical College, Tongji Hospital, Huazhong University of Science and Technology, 1095 Jiefang Avenue, Wuhan, 430030 Hubei China

**Keywords:** Blood biomarkers, Post-stroke depression, Sex difference

## Abstract

**Background:**

Post-stroke depression (PSD) is one of the most common neuropsychiatric complications after stroke. Studies on the underlying mechanisms and biological markers of sex differences in PSD are of great significance, but there are still few such studies. Therefore, the main objective of this study was to investigate the association of biomarkers with PSD at 3 months after minor stroke in men and women.

**Methods:**

This was a prospective multicenter cohort study that enrolled 530 patients with minor stroke (males, 415; females, 115). Demographic information and blood samples of patients were collected within 24 h of admission, and followed up at 3 months after stroke onset. PSD was defined as a depressive disorder due to another medical condition with depressive features, major depressive-like episode, or mixed-mood features according to the Diagnostic and Statistical Manual of Mental Disorders, 5th edition (DSM-V). Univariate analysis was performed using the chi-square test, Mann–Whitney U test, or t-test. Partial least-squares discriminant analysis (PLS-DA) was used to distinguish between patients with and without PSD. Factors with variable importance for projection (VIP) > 1.0 were classified as the most important factors in the model segregation.

**Results:**

The PLS-DA model mainly included component 1 and component 2 for males and females. For males, the model could explain 13% and 16.9% of the variables, respectively, and 29.9% of the variables in total; the most meaningful predictors were exercise habit and fibrinogen level. For females, the model could explain 15.7% and 10.5% of the variables, respectively, and 26.2% of the variables in total; the most meaningful predictors in the model were brain-derived neurotrophic factor (BDNF), magnesium and free T3. Fibrinogen was positively correlated with the Hamilton Depression Scale-17 items (HAMD-17) score. BDNF, magnesium, and free T3 levels were negatively correlated with the HAMD-17 score.

**Conclusions:**

This was a prospective cohort study. The most important markers found to be affecting PSD at 3 months were fibrinogen in males, and free T3, magnesium, and BDNF in females.

**Trial registration:**

ChiCTR-ROC-17013993.

**Supplementary Information:**

The online version contains supplementary material available at 10.1186/s12888-022-03805-6.

## Background

Post-stroke depression (PSD) is one of the most common neuropsychiatric complications after stroke [1]. The overall prevalence of PSD is approximately 29%, with little change over time after stroke: 28% at 1 month, 31% at 1 to 6 months, 33% at 6 months to 1 year, and 25% after 1 year [2]. PSD is associated with a reduced quality of life and poor functional recovery after stroke [3]. In addition, 12.2% of stroke patients had suicidal thoughts, and 11.3% had suicidal plans due to mood disorders [4–6].

The development of PSD appears to be mediated by multiple overlapping social, psychological, functional, and biological factors [7]. Therefore, it is necessary to study the occurrence and development mechanisms of PSD from various perspectives. Identification of blood biomarkers can improve the accuracy of PSD diagnosis and facilitate early intervention. Previous studies have reported that bilirubin [8], blood lipids [9], electrolyte levels [10], homocysteine [11], hypersensitive C-reactive protein (CRP) [12], fibrinogen [10], cytokines [13, 14] and various hormones [15, 16] have an impact on the occurrence and development of PSD.

Previous studies have reported that the prevalence of PSD in males may be greater than [17], equal to [18], or less than [19] that in females. Different factors might have different effects on the onset of depression in men and women. Studies on the underlying mechanisms and biological markers of sex differences in PSD are of great significance for the diagnosis and treatment of PSD, but there are still few such studies. In recent years, the mechanism of PSD in minor strokes has attracted much attention [20]. Previous studies have found that higher National Institutes of Health Stroke Scale (NIHSS) scores are associated with the development of PSD [21]. However, some patients with minor strokes are still prone to develop PSD symptoms even with less neurological deficits or full recovery [22]. Therefore, there may be different biological factors involved in the development of PSD in patients with minor strokes as compared to an adaptation disorder due to a more severe stroke. The main objective of this study was to investigate the association of the above-mentioned biomarkers with PSD at 3 months after minor stroke in men and women.

## Methods

All procedures described in this manuscript were approved by the Ethics Committee of Tongji Medical College, Huazhong University of Science and Technology (Approval No: TJ-IRB20171108), and the study is a registered clinical trial (registration number: ChiCTR-ROC 17,013,993). A total of 530 patients with minor stroke hospitalized in the Department of Neurology of Tongji Hospital, Wuhan First Hospital, and Wuhan Central Hospital in Wuhan, Hubei Province, China, were enrolled from May 2018 to August 2019. The study was conducted according to the Helsinki Declaration, and all participants gave written informed consent.

### Subjects

The inclusion criteria for this study were as follows: (1) age ≥ 18 years; (2) hospitalization within 7 days after stroke onset (including hemorrhagic and ischemic stroke); (3) stroke confirmed by computed tomography (CT) or magnetic resonance imaging (MRI) scan; (4) minor stroke: a NIHSS score ≤ 3 points; (5) blood samples were collected within 24 h after admission; and (6) informed consent signed by patients or family members. Exclusion criteria were: (1) brain dysfunction caused by non-vascular diseases such as brain trauma, brain tumor, and metastatic brain tumor; (2) a history of anxiety, depression, or other mental diseases or taking related drugs; (3) aphasia, blindness, deafness, and cognitive dysfunction; (4) subarachnoid hemorrhage; and (5) poor compliance or cannot complete the experiment [23].

### Depression assessment

Demographic and medical history information of patients was collected within 24 h of admission, including age, height, weight, stroke type, education level, smoking history, drinking history, sleeping time, diabetes mellitus, hypertension, hyperlipidemia, coronary heart disease (CHD), stroke history, and exercise habits. The NIHSS Mini-Mental State Examination (MMSE) and Hamilton Depression Scale-17 items (HAMD-17) were assessed by two qualified and formally trained doctors (C.P. and W.S) at admission and at 3 months after stroke onset. PSD was diagnosed by a psychiatrist according to the diagnostic criteria for PSD in the Diagnostic and Statistical Manual of Mental Disorders, 5th edition (DSM-V) (i.e., depressive disorder due to another medical condition with depressive features, major depressive-like episode, or mixed-mood features [24]), and the HAMD-17 score was greater than 7 after stroke onset.

### Blood collection

Venous blood samples were collected in the early morning of the second day (within 24 h of admission) and sent to the laboratory for testing. The serological indicators tested in the laboratory included total bilirubin, direct bilirubin, indirect bilirubin, total cholesterol, triglyceride, high-density lipoprotein (HDL), low-density lipoprotein (LDL), potassium, sodium, chlorine, calcium, phosphorus, magnesium, homocysteine, CRP, thyroid stimulating hormone (TSH), free triiodothyronine (T3), free tetraiodothyronine (T4), fibrinogen, D-dimer, glycosylated hemoglobin (HbA1C), prolactin (only females), estradiol (only females), testosterone (only males), interleukin 1β, interleukin 6, interleukin 10, interleukin 18, tumor necrosis factor-α (TNF-α), brain-derived neurotrophic factor (BDNF), interferon-γ (INF- γ), fasting C peptide, cortisol, and adrenocorticotrophic hormone (ACTH).

### Statistical analysis

The Statistical Program for Social Sciences (SPSS) statistical software (version 25, Chicago, IL, USA) was used for the data analysis. Continuous variables were represented by medium and inter-quartile range (IQR) or mean ± standard deviation and were compared using the Mann–Whitney U test or T test (when the data were normally distributed). Categorical variables were represented by the number of cases and percentages, and analyzed using the chi-square test. To identify all potentially significant variables, variables with *p* < 0.5 were selected for multivariate logistic regression analysis. Differences were considered statistically significant at a *p* < 0.05. Consistency between observers, as measured by the HAMD-17 score, was determined using the intra-group correlation coefficient (ICC).

We used partial least-squares discriminant analysis (PLS-DA) to explore the relationship between serological indicators and PSD [25]. PLS is an exploratory multivariate analysis technique that models the relationship between a set of predictive and response variables based on a set of mutually orthogonal potential factors or PLS components [26]. It does not require a distribution hypothesis; therefore, it is also suitable for the analysis of skewness in the data distribution.

The models were conducted using “ropls” package of R software (v4.0.0; http://www.r-project.org/). Here, we modeled the HAMD-17 score as the response variable Y. The predictor variables, X, comprised 13 demographic variables and 34 serological indicators. Data from all patients were included, and missing data were imputed by multiple imputation. Since many independent variables may be correlated, other statistical methods (such as linear regression) will be ineffective. In this case, PLS is an ideal statistical analysis technique.

A logarithmic transformation (Log10) was used for fold-change values for better symmetry between the distribution curves and the autoscaling technique for data standardization. The model was constructed in two steps. First, the partial least squares method was used to determine the optimal number of factors to be included in the PLS-DA model. Second, PLS-DA was used to distinguish between PSD and non-PSD patients. Factors with variable importance for projection (VIP) > 1.0, were classified as the most important factors in the model segregation. Reduced PLS-DA models were tested with these factors until a model with greater predictive capacity using the fewest possible variables was obtained [25].

## Results

**Fig. 1:**
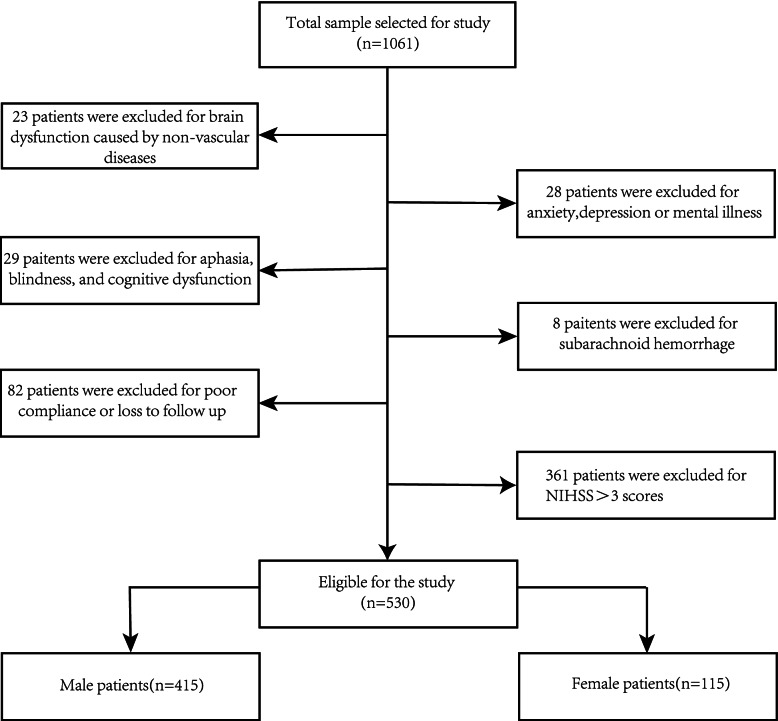
A flow diagram drawn according to inclusion and exclusion criteria

The initial study included 1061 stroke patients, and 530 patients (415 men and 115 women) were included after screening for inclusion and exclusion criteria to exclude 531 patients (Fig. 1). The proportion of PSD at 3 months was 30.1% in men and 38.3% in women. The HAMD-17 score (ICC = 0.92, 95% CI: 0.79–0.97) had high inter-observer consistency.

A univariate analysis of the demographics of both males and females found that males were more obese than females (*p* < 0.001), and had a higher proportion of medium to high education level (*p* < 0.001), smoking history (*p* < 0.001), drinking history (*p* < 0.001), and sleep time below than 5 h (*p* = 0.038). There were no significant differences in other demographic variables between males and females (Table 1).

**Table 1:** Univariate regression analysis of male and female demographic data

Variable	Female(*n* = 115)	Male(*n* = 415)	*p* value
Age, mean ± SD	58.8 ± 12.3	58.0 ± 11.5	0.341
BMI, mean ± SD	23.4 ± 3.2	24.6 ± 3.1	< 0.001
Stroke type			0.066
Infarction, n(%)	98(85.2)	378(91.1)	
Hemorrhage, n(%)	17(14.8)	37(8.9)	
Education level			< 0.001
Low, n(%)	52(45.2)	80(19.3)	
Medium, n(%)	49(42.6)	243(58.6)	
High, n(%)	14(14)	92(22.2)	
Smoking history, n(%)	12(10.4)	312(75.2)	< 0.001
Drinking history, n(%)	8(7.0)	140(33.7)	< 0.001
Sleeping time < 5 h, n(%)	18(15.7)	103(24.8)	0.038
Diabetes Mellitus, n(%)	24(20.9)	107(25.8)	0.280
Hypertension, n(%)	66(57.4)	238(57.3)	0.994
Hyperlipidemia, n(%)	21(18.3)	87(21.0)	0.524
CHD, n(%)	9(7.8)	38(9.2)	0.657
Stroke history, n(%)	19(16.5)	87(21.0)	0.292
Exercise habit, n(%)	45(39.1)	171(41.2)	0.689

*BMI* body mass index, *CHD* coronary heart disease

**Fig. 2:**
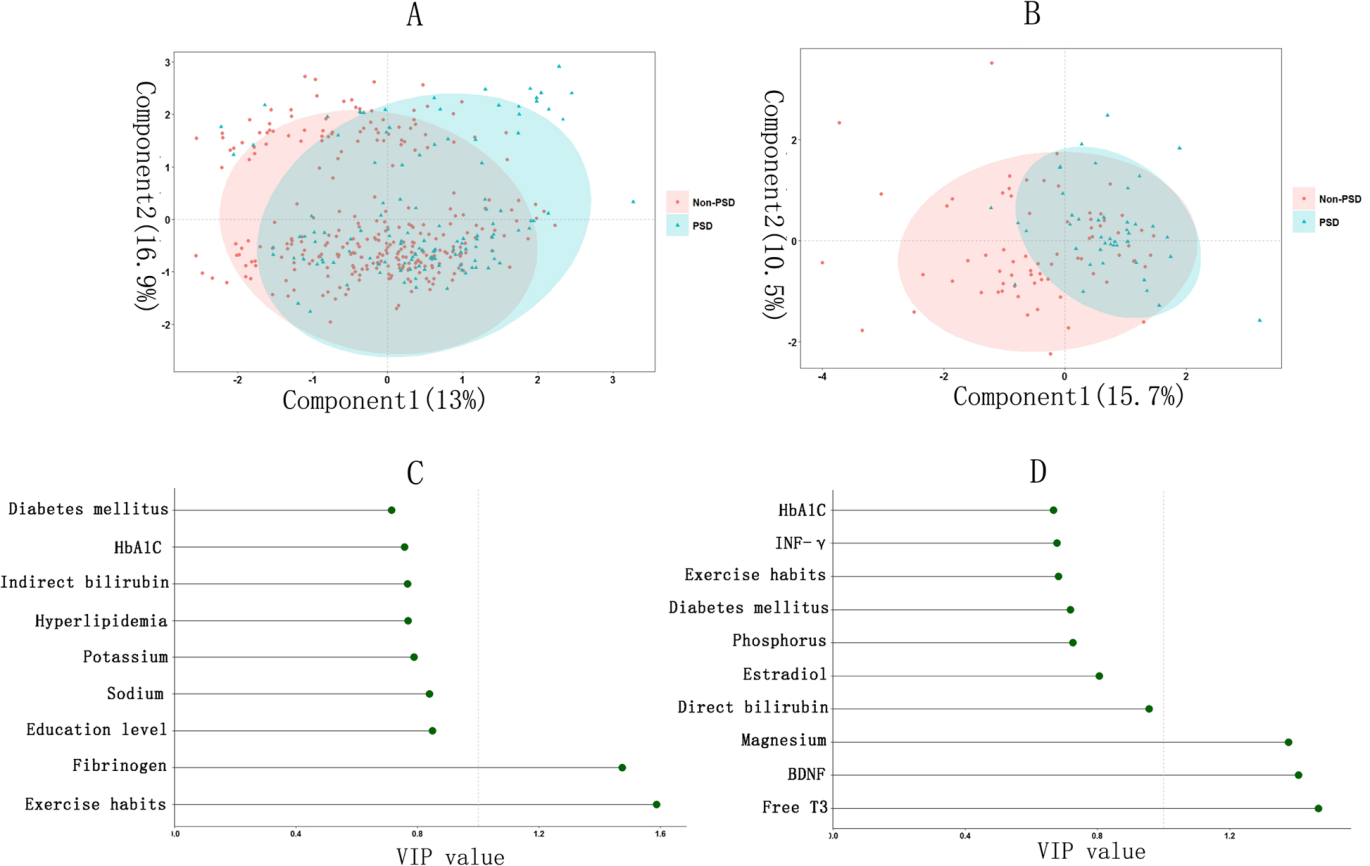
**A** Partial least-squares discriminant analysis(PLS-DA) model of male of PSD at 3 months; (**B**) PLS-DA model of female of PSD at 3 months; (**C**) Variable importance for projection(VIP) values of factors included in male PLS-DA model; (**D**) VIP values of factors included in female PLS-DA model; HbA1C: glycosylated hemoglobin A1c; INF-γ: interferon-γ; BDNF: brain derived neurotrophic factor

According to the PLS method, the optimal number of factors included in the male PLS-DA model was nine (Additional file 1). The PLS-DA model for males mainly included component 1 and component 2, which explained 13% and 16.9% of variables, respectively, and 29.9% of variables in total. The most meaningful predictors in the model were exercise habit and fibrinogen level (VIP value > 1) (Fig. 2). The optimal number of factors included in the female PLS-DA model was 10 (Additional file 1). The PLS-DA model for females also mainly included component 1 and component 2, which explained 15.7% and 10.5% of the variables, respectively, and 26.2% of the variables in total. The most meaningful predictors in the model were BDNF, magnesium, and free T3 (VIP value > 1) (Fig. 2).

**Fig. 3:**
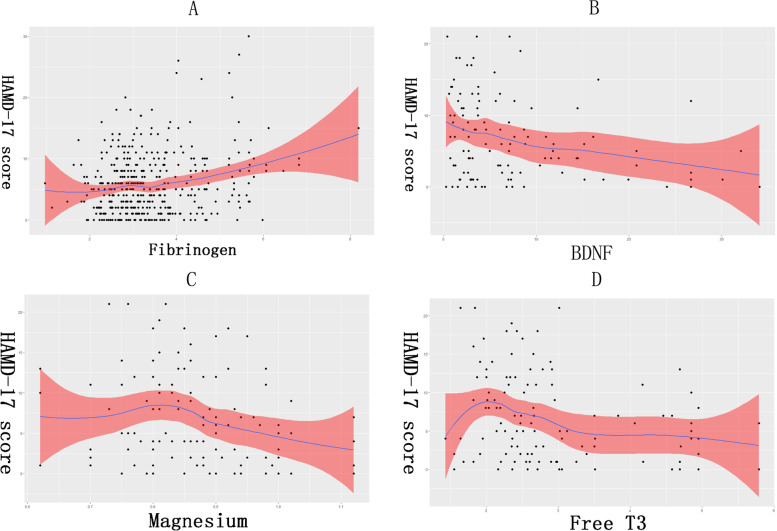
**A** The curve and standard error about fibrinogen at admission and HAMD-17 score at 3 months of stroke; (**B**) The curve and standard error about BDNF at admission and HAMD-17 score at 3 months of stroke; (**C**) The curve and standard error about magnesium at admission and HAMD-17 score at 3 months of stroke; (**D**) The curve and standard error about free T3 at admission and HAMD-17 score at 3 months of stroke. BDNF: brain derived neurotrophic factor

Fibrinogen (SE [Standard errors] = 0.230, β = 1.030) was important in predicting PSD at 3 months in males, fitting the relationship between fibrinogen and the HAMD-17 score and showing standard error (Fig. 3). Fibrinogen was positively correlated with HAMD-17 score. BDNF (SE = 0.067, β = -0.208), magnesium (SE = 5.120, β = -10.571), and free T3 (SE = 0.499, β = -1.409) were important in predicting PSD at 3 months in females, fitting the relationship between BDNF, magnesium, or free T3 and HAMD-17 scores and showing standard error (Fig. 3). BDNF, magnesium, and free T3 levels were negatively correlated with HAMD-17 score.

In addition, we determined whether the results of traditional univariate and multivariate logistic regression analyses were consistent with the PLS-DA analysis. The results of the univariate analysis are presented in Additional file 1: Table S1. In the multivariate logistic regression analysis, it remained that BDNF (*p* = 0.029, OR = 0.916, 95%CI: 0.846–0.991), FT3 (*p* = 0.004, OR = 0.463, 95%CI: 0.273–0.784) and magnesium (*p* = 0.003, OR = 0.001, 95%CI: 0.001–0.071) were significantly different between the women in the PSD and non-PSD groups, whereas exercise habit (*p* = 0.001, OR = 0.462, 95%CI: 0.292–0.731), fibrinogen (*p* = 0.004, OR = 1.342, 95%CI: 1.096–1.643) and FT4 (*p* = 0.049, OR = 1.039, 95%CI: 1.004–1.643) were significantly different between men in the PSD and non-PSD groups (Table 2). After multiple comparisons, BDNF, FT3, magnesium and fibrinogen remained significantly different, consistent with the results of the PLS-DA analysis (Tables 3 and 4).

**Table 2:** Multivariate analysis of clinical variables and blood biomarkers variables in PSD and non-PSD of male and female

Variables	β	*p*	OR	95%CI
**Female**				
Magnesium	-7.878	0.003	0.001	0.001–0.071
FT3	-0.770	0.004	0.463	0.273–0.784
BDNF	-0.088	0.029	0.916	0.846–0.991
**Male**				
Exercise habit	-0.772	0.001	0.462	0.292–0.731
Fibrinogen	0.294	0.004	1.342	1.096–1.643
FT4	0.038	0.049	1.039	1.004–1.643

*FT4* free tetraiodothyronine, *FT3* free triiodothyronine, *BDNF* brain-derived neurotrophic factor

**Table 3:** The multiple comparisons of magnesium, FT3 and BDNF in female

Variables	Model 1				Model 2				Model 3			
	β	*p*	OR	95%CI	β	*p*	OR	95%CI	β	*p*	OR	95%CI
Magnesium	-7.696	0.011	0.001	0.001–0.166	-	-	-	-	-	-	-	-
FT3	-	-	-	-	-0.886	0.004	0.412	0.227–0.747	-	-	-	-
BDNF	-	-	-	-	-	-	-	-	-0.135	0.012	0.874	0.787–0.971
Age	0.001	0.981	1.000	0.961–1.041	0.021	0.300	1.022	0.981–1.064	0.010	0.605	1.001	0.971–1.052
BMI	-0.022	0.781	0.979	0.840–1.140	-0.049	0.527	0.952	0.817–1.109	-0.020	0.805	0.981	0.839–1.146
Smoking history	-1.663	0.068	0.190	0.032–1.133	-1.128	0.227	0.324	0.052–2.018	-1.383	0.134	0.251	0.041–1.534
Hypertension	0.795	0.146	2.215	0.758–6.470	0.862	0.120	2.367	0.799–7.013	0.778	0.158	2.176	0.740–6.401
Hyperlipidemia	-0.509	0.458	0.601	0.157–2.302	-0.962	0.192	0.382	0.090–1.621	-0.496	0.471	0.609	0.158–2.342
Exercise habit	-1.124	0.038	0.325	0.112–0.941	-1.052	0.064	0.394	0.115–1.061	-1.012	0.072	0.363	0.121–1.095
Triglyceride	0.063	0.740	1.065	0.735–1.542	0.032	0.859	1.032	0.727–1.466	-0.075	0.683	0.928	0.648–1.329
HDL	-1.237	0.180	0.290	0.048–1.771	-2.051	0.034	0.129	0.019–0.857	-1.894	0.033	0.150	0.026–0.858
Potassium	-0.039	0.920	0.961	0.445–2.080	0.069	0.859	1.072	0.498–2.306	-0.065	0.864	0.937	0.446–1.967
Calcium	-3.501	0.061	0.030	0.001–1.167	-4.531	0.038	0.011	0.001–0.771	-1.471	0.442	0.230	0.005–9.792
Phosphorus	-0.139	0.909	0.870	0.081–9.343	0.575	0.667	1.778	0.129–24.474	-1.039	0.376	0.354	0.036–3.523
D-dimer	-0.528	0.249	0.590	0.241–1.446	-0.909	0.049	0.403	0.163–0.997	-0.869	0.063	0.420	0.168–1.047
HbA1C	-0.293	0.063	0.746	0.548–1.016	-0.141	0.306	0.868	0.663–1.138	-0.169	0.248	0.844	0.634–1.125
Estradiol	0.006	0.110	1.006	0.999–1.013	0.008	0.034	1.008	1.001–1.006	0.007	0.052	1.007	1.000–1.015
Interleukin 6	0.001	0.994	1.000	0.976–1.025	-0.006	0.586	0.994	0.972–1.016	0.001	0.989	1.000	0.977–1.023
Interleukin 10	0.004	0.165	1.004	0.998–1.011	0.003	0.391	1.003	0.996–1.010	0.002	0.431	1.002	0.996–1.009
Cortisol	0.003	0.911	1.003	0.956–1.052	-0.002	0.961	0.998	0.919–1.083	-0.001	0.986	0.999	0.935–1.068
ACTH	0.002	0.852	1.002	0.981–1.024	-0.003	0.785	0.997	0.975–1.019	-0.006	0.629	0.994	0.972–1.017

*BMI* body mass index, *HDL* high-density lipoprotein, *FT3* free triiodothyronine, *BDNF* brain-derived neurotrophic factor, *ACTH* brain-derived neurotrophic factor

**Table 4:** The multiple comparisons of exercise habit, fibrinogen and FT4 in male

Variables	Model 1				Model 2				Model 3			
	β	*p*	OR	95%CI	β	*p*	OR	95%CI	β	*p*	OR	95%CI
Exercise habit	-0.817	0.002	0.442	0.266–0.734	-	-	-	-	-	-	-	-
Fibrinogen	-	-	-	-	0.324	0.011	1.383	1.076–1.777	-	-	-	-
FT4	-	-	-	-	-	-	-		0.042	0.051	1.043	1.006–1.082
Age	0.010	0.382	1.010	0.988–1.033	0.002	0.868	1.002	0.980–1.024	0.006	0.599	1.006	0.984–1.028
Stroke type	0.580	0.161	1.786	0.794–4.017	0.525	0.204	1.690	0.753–3.795	0.711	0.087	2.036	0.902–4.595
Education level												
Low		Rf	Rf	Rf		Rf	Rf	Rf		Rf	Rf	Rf
Medium	-0.342	0.253	0.710	0.394–1.28	-0.388	0.196	0.679	0.377–1.221	-0.390	0.190	0.677	0.378–1.214
High	-0.578	0.131	0.561	0.265–1.187	-0.723	0.055	0.485	0.232–1.014	-0.773	0.040	0.462	0.220–0.967
Smoking history	-0.291	0.288	0.748	0.438–1.278	-0.381	0.161	0.683	0.401–1.163	-0.343	0.206	0.710	0.417–1.208
Diabetes Mellitus	0.882	0.010	2.416	1.234–4.729	0.767	0.024	2.153	1.108–4.184	0.815	0.016	2.260	1.166–4.377
Hyperlipidemia	-0.492	0.118	0.611	0.329–1.134	-0.595	0.061	0.551	0.296–1.027	-0.512	0.105	0.600	0.323–1.112
Stroke history	-0.450	0.146	0.638	0.348–1.169	-0.406	0.188	0.666	0.364–1.219	-0.377	0.220	0.686	0.376–1.253
Direct bilirubin	0.109	0.149	1.115	0.962–1.292	0.089	0.236	1.093	0.943–1.266	0.078	0.296	1.081	0.934–1.251
Indirect bilirubin	-0.064	0.035	0.938	0.883–0.996	-0.049	0.104	0.952	0.898–1.010	-0.062	0.042	0.940	0.886–0.998
Triglyceride	-0.078	0.375	0.925	0.777–1.100	-0.024	0.782	0.976	0.822–1.158	-0.054	0.546	0.947	0.794–1.130
Potassium	-0.364	0.306	0.695	0.346–1.395	-0.378	0.284	0.685	0.343–1.368	-0.361	0.310	0.697	0.347–1.399
Sodium	0.071	0.253	1.073	0.951–1.212	-0.085	0.160	1.089	0.967–1.227	0.090	0.147	1.094	0.969–1.235
Chlorine	-0.061	0.201	0.941	0.856–1.033	-0.053	0.259	0.948	0.864–1.040	-0.061	0.204	0.941	0.857–1.033
Calcium	-1.162	0.319	0.313	0.032–3.076	-1.110	0.336	0.330	0.034–3.167	-1.142	0.314	0.319	0.035–2.947
Phosphorus	0.792	0.252	2.208	0.570–8.553	0.724	0.291	2.063	0.538–7.916	0.898	0.187	2.455	0.646–9.333
Magnesium	-0.262	0.862	0.770	0.040–14.763	-0.216	0.886	0.806	0.043–15.290	0.141	0.924	1.151	0.063–21.116
CRP	-0.006	0.406	0.994	0.981–1.008	-0.010	0.198	0.990	0.975–1.005	-0.003	0.690	0.997	0.984–1.001
D-dimer	0.060	0.378	1.062	0.929–1.215	0.071	0.309	1.074	0.936–1.231	0.065	0.342	1.067	0.933–1.220
HbA1C	-0.169	0.105	0.844	0.688–1.036	-0.195	0.062	0.823	0.671–1.010	-0.184	0.076	0.832	0.679–1.020
Testosterone	-0.006	0.930	0.994	0.869–1.137	-0.003	0.961	0.997	0.871–1.140	-0.003	0.960	0.997	0.871–1.140
Interleukin 6	-0.001	0.822	0.999	0.995–1.004	0.001	0.899	1.001	0.994–1.005	-0.001	0.770	0.999	0.995–1.004
TNF-α	-0.03	0.080	0.997	0.993–1.000	-0.003	0.072	0.997	0.993–1.000	-0.003	0.074	0.997	0.993–1.000
Interferon-γ	0.001	0.866	1.000	0.997–1.003	0.001	0.594	1.001	0.998–1.004	0.001	0.524	1.001	0.998–1.004
Fasting C peptide	0.143	0.091	1.154	0.978–1.363	0.110	0.193	1.116	0.946–1.317	0.114	0.169	1.121	0.953–1.318
Cortisol	0.019	0.473	1.019	0.967–1.075	0.004	0.883	1.004	0.953–1.058	0.016	0.547	1.016	0.965–1.070
ACTH	0.002	0.636	1.002	0.993–1.012	0.002	0.715	1.002	0.992–1.011	0.001	0.837	1.001	0.991–1.082

*CRP* hypersensitive C-reactive protein, *FT4* free tetraiodothyronine, *HbA1C* glycosylated hemoglobin, *TNF-α* tumor necrosis factor-α, *ACTH* brain-derived neurotrophic factor

## Discussion

In this study, we found that fibrinogen may be a predictive blood biomarker for PSD at 3 months in males, and free T3, magnesium, and BDNF may be predictive blood biomarkers for PSD at 3 months in females. This suggests that clinicians should consider the different effects of the above blood biomarkers on men and women in the screening, diagnosis, and treatment of PSD, in order to adopt different preventive or therapeutic measures.

In this study, the proportion of PSD at 3 months in females was higher than that in males (38.3% vs 30.1%). Possible reasons for this difference are as follows: (1) Testosterone is a neuroactive steroid hormone that regulates many neurotransmitters and/or their associated receptors, such as γ-aminobutyric acid (GABA), dopamine and serotonin (5-HT), which may underlie its protective effect against depressive symptoms [27]. (2) Studies of functional connections between the dorso-medial prefrontal cortex and the right amygdala suggest that males process negative emotions more through rational assessment than purely emotional responses [28–30]. Therefore, males may be able to better deal with negative emotions when a stroke occurs. (3) Expression of some genes associated with depressive symptoms is sex-specific [31]. For example, downregulation of Dusp6, a female-specific hub gene in the major depressive disorder network, in the mouse prefrontal cortex mimics stress susceptibility in females, but not males, by increasing ERK signaling and pyramidal neuron excitability [32].

Previous studies have found a link between elevated fibrinogen levels and depressive symptoms [33]. Our study found that higher fibrinogen levels at admission increased the risk of PSD at 3 months in men. Fibrinogen has been shown to stimulate the synthesis of pro-inflammatory cytokines such as TNF-α and interleukin-6 by peripheral blood mononuclear cells, thereby increasing the levels of pro-inflammatory cytokines [34]. These pro-inflammatory cytokines activate indolamine-2,3-dioxygenase, which degrades tryptophan to kynurenine [35, 36]. Since tryptophan is a precursor of serotonin, a decrease in tryptophan concentration leads to a decrease in serotonin synthesis, which may contribute to the development of depression [37].

Magnesium is an important micronutrient essential for the synthesis of many biochemical substances and normal physiological activities of the human body [38]. Magnesium deficiency has been shown to cause changes in central nervous system function, particularly glutamate transmission in the limbic system and cerebral cortex, which play an important role in the pathogenesis of depression [39, 40]. In addition, magnesium prevents overactivation of the hypothalamic–pituitary–adrenal axis by reducing ACTH release and regulating adrenal cortex sensitivity to ACTH [41]. Dysregulation of the hypothalamic axis in adults is closely associated with depression, and elevated cortisol levels and dysregulation of hypothalamic activity are common in depressed individuals [42, 43].

Free T3 plays an important role in the endocrine system and the basic metabolism of the human body. Thyroid hormone affects the functions of serotonin and catecholamines in the brain, and plasma serotonin levels are positively correlated with T3 concentration [44]. Free T3 may enhance the role of serotonin in the brain by regulating the transcription of serotonin receptors by altering the mRNA encoding of 5-HT1A (5-hydroxytryptamine)- and 5-HT1B receptors [45]. In addition, free T3 as an active thyroid hormone in the brain decreases the risk of PSD, which may be due to its ability to promote neural protection [46] and nerve regeneration [47], or to an effect of free T3 itself on depression.

BDNF is an important neurotrophic factor that binds to the tropomyosin kinase B (TrkB) receptor and plays an important role in synaptic development and plasticity [48]. BDNF and its receptor TrkB are expressed in mesolimbic dopamine (DA) circuits that project midbrain DA neurons in the ventral tegmental region (VTA) to the basal forebrain nucleus accumbens (NAc) [49, 50]. BDNF-TrkB and DA signals in the mesolimbic circuit play key roles in stress-related and reward-related behaviors [51, 52]. Mesolimbic BDNF-TrkB signal transduction is also implicated in the pathophysiology of depressive symptoms [53, 54], and dysregulation of the mesolimbic DA system is associated with depression-related behaviors [55].

Despite the previously described advantages of PLS-DA, it did not discriminate well between PSD and non-PSD patients in our study. In the reported literature, PLS-DA is primarily used for proteomics analyses, which generally requires models with an accuracy of 50% or more, or at least 40% [25, 56]. In clinical studies, model accuracy greater than 20% is acceptable due to large and uncontrollable individual differences [57–59]. As mentioned earlier, the development of PSD appears to be mediated by multiple overlapping social, psychological, functional, and biological factors [7]. Therefore, we used the PLS-DA method with the main purpose of screening for markers with diagnostic and therapeutic significance, with the expectation that they will be suggestive for subsequent studies and clinicians.

This study had several advantages. First, demographic indicators were included in the analysis as covariates to ensure the stability of the results as far as possible. Second, through the analysis of male and female groups, it was found that the blood markers affecting male and female PSD at 3 months were different. Third, the PLS-DA method was used to screen blood markers, which does not require the normality of the data distribution.

As for the study limitations, first, the study had a small sample size, with a total sample of 530 patients and 115 females. Second, excluding people with aphasia, blindness, deafness, and cognitive impairment may have skewed the results. Third, we did not include stroke volume in the analysis, which is an important factor influencing the occurrence of PSD [60]. In future studies, we will use artificial intelligence, the brain atlas, and voxel-based analyses to analyze the relationship between stroke volume and PSD. Finally, the follow-up time of the study was short, and a longer follow-up time may be more helpful in observing the occurrence and development of depression in stroke patients.

## Conclusion

This was a prospective cohort study. Through the analysis of males and females, it was found that the most important marker affecting PSD at 3 months in males was fibrinogen, and the most important markers affecting PSD at 3 months in males were free T3, magnesium, and BDNF.

## Supplementary Information

**Additional file 1.**

## Data Availability

The datasets analysed during the current study available from the corresponding author on reasonable request. Requests to access the datasets should be directed to zhouzhu@hust.edu.cn.
