# Supplementary material for: Blood biomarkers of post-stroke depression after minor stroke at three months in males and females
Source: BMC Psychiatry. 2022 Mar 3;22:162. doi: 10.1186/s12888-022-03805-6 (PMC8896360; doi:10.1186/s12888-022-03805-6)
Supplement: Supplementary file 1 — Additional file 1. [file 12888_2022_3805_MOESM1_ESM.docx]

**
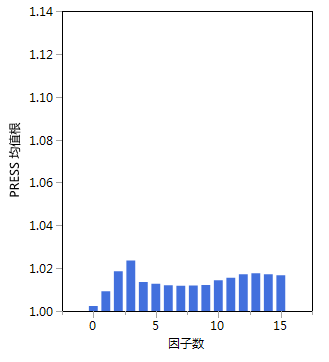
**

Figure 4. The optimal number of factors included in the PLS-DA model of male. The X-axis represents the number of factors and the Y-axis represents the mean root value.


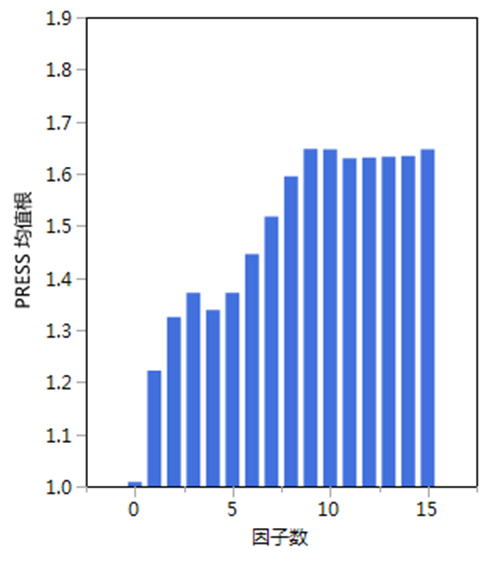


Figure 5. The optimal number of factors included in the PLS-DA model of female. The X-axis represents the number of factors and the Y-axis represents the mean root value.

Table 2. Univariate analysis of clinical variables and blood biomarkers in PSD and non-PSD of male and female

| Variable | Female | | | Male | | |
| --- | --- | --- | --- | --- | --- | --- |
|  | PSD(n=44) | Non-PSD(n=71) | p value | PSD(n=125) | Non-PSD(n=290) | p value |
| Age, mean±SD | 58.3±11.0 | 59.2±13.0 | 0.517 | 58.0±11.5 | 58.1±11.5 | 0.973 |
| BMI, mean±SD | 23.1±3.4 | 23.5±3.0 | 0.328 | 24.8±2.8 | 24.5±3.2 | 0.531 |
| Stroke type  Infarction, n(%)  Hemorrhage, n(%) | 38(86.4)  6(13.6) | 60(84.5)  11(15.4) | 0.785 | 110(88.0)  15(12.0) | 268(92.4)  22(7.6) | 0.148 |
| Education level  Low, n(%)  Medium, n(%)  High, n(%) | 22(50.0)  18(40.9)  4(9.1) | 30(42.3)  31(43.7)  10(14.1) | 0.617 | 30(24.0)  72(57.6)  23(18.4) | 50(17.2)  171(59.0)  69(23.8) | 0.198 |
| Smoking history, n(%) | 3(6.8) | 9(12.7) | 0.493 | 90(72.0) | 222(76.6) | 0.325 |
| Drinking history, n(%) | 3(6.8) | 5(7.0) | 1.000 | 42(33.6) | 98(33.8) | 0.970 |
| Sleeping time＜5h, n(%) | 7(15.9) | 11(15.5) | 0.952 | 32(25.6) | 71(24.5) | 0.809 |
| Diabetes Mellitus, n(%) | 9(20.5) | 15(21.1) | 0.931 | 35(28.0) | 72(24.8) | 0.498 |
| Hypertension, n(%) | 27(61.4) | 39(54.9) | 0.498 | 70(56.0) | 168(57.9) | 0.715 |
| Hyperlipidemia, n(%) | 6(13.6) | 15(21.1) | 0.312 | 20(16.0) | 67(23.1) | 0.103 |
| CHD, n(%) | 3(6.8) | 6(8.5) | 1.000 | 12(9.6) | 26(9.0) | 0.837 |
| Stroke history, n(%) | 6(13.6) | 13(18.3) | 0.512 | 22(17.6) | 65(22.4) | 0.269 |
| Exercise habit, n(%) | 14(31.8) | 31(43.7) | 0.206 | 36(28.8) | 135(46.6) | 0.001 |
| NIHSS score, median(IQR) | 1.5(1-3) | 2(1-2) | 0.979 | 1(1-2) | 1(1-2) | 0.434 |
| Total bilirubin, median(IQR) | 9.7(6.8-16.6) | 10.6(7.5-15.0) | 0.874 | 11.8(8.8-16.4) | 12.8(8.9-16.5) | 0.538 |
| Direct bilirubin, median(IQR) | 3.3(2.3-5.4) | 3.2(2.1-5.5) | 0.713 | 4.0(2.7-5.4) | 3.6(2.4-4.8) | 0.119 |
| Indirect bilirubin, median(IQR) | 6.0(4.0-10.4) | 7.2(4.3-10.4) | 0.637 | 6.9(5.0-10.3) | 8.1(5.5-11.8) | 0.040 |
| Total cholesterol, median(IQR) | 4.2(3.7-4.9) | 4.4(3.6-5.1) | 0.613 | 4.0(3.3-4.8) | 3.9(3.3-4.8) | 0.776 |
| Triglyceride, median(IQR) | 1.1(0.7-1.7) | 1.4(0.8-2.3) | 0.137 | 1.3(0.9-1.9) | 1.5(1.0-2.0) | 0.246 |
| HDL, median(IQR) | 1.1(0.9-1.2) | 1.1(0.9-1.3) | 0.304 | 0.9(0.8-1.1) | 0.9(0.8-1.3) | 0.612 |
| LDL, median(IQR) | 2.8(2.2-3.4) | 2.8(2.1-3.5) | 0.899 | 2.5(2.0-3.3) | 2.5(1.9-3.1) | 0.683 |
| Potassium, median(IQR) | 3.8(3.6-4.0) | 3.8(3.7-4.2) | 0.300 | 3.8(3.6-4.1) | 3.9(3.7-4.2) | 0.112 |
| Sodium, median(IQR) | 141.2(139.4-143.8) | 141.1(139.1-142.5) | 0.592 | 141.1(139.7-142.3) | 140.7(138.5-141.9) | 0.030 |
| Chlorine, median(IQR) | 102.9(101.2-106.7) | 103.1(100.8-105.9) | 0.590 | 102.9(100.6-105.0) | 103.2(100.7-105.3) | 0.254 |
| Calcium, median(IQR) | 2.2(2.1-2.3) | 2.3(2.2-2.4) | 0.016 | 2.3(2.2-2.3) | 2.3(2.2-2.3) | 0.266 |
| Phosphorus, median(IQR) | 1.1(0.9-1.2) | 1.1(1.0-1.3) | 0.059 | 1.0(0.9-1.1) | 1.0(0.9-1.1) | 0.123 |
| Magnesium, median(IQR) | 0.8(0.8-0.8) | 0.9(0.8-1.0) | 0.007 | 0.9(0.8-0.9) | 0.8(0.8-0.9) | 0.310 |
| Homocysteine, median(IQR) | 11.9(9.3-14.4) | 12.0(9.2-15.1) | 0.649 | 15.0(12.3-20.0) | 15.0(12.0-20.5) | 0.876 |
| CRP, median(IQR) | 1.3(0.5-3.3) | 1.9(0.3-6.0) | 0.963 | 1.9(1.0-4.7) | 1.6(0.6-4.8) | 0.264 |
| TSH, median(IQR) | 2.2(1.1-3.4) | 1.9(0.7-3.6) | 0.651 | 1.6(0.9-2.8) | 1.7(0.9-2.8) | 0.986 |
| FT3, median(IQR) | 2.3(2.0-2.7) | 2.7(2.4-3.8) | 0.001 | 2.7(2.4-3.9) | 2.8(2.4-3.2) | 0.655 |
| FT4, median(IQR) | 1.1(0.9-1.2) | 1.0(0.8-1.2) | 0.547 | 1.0(0.9-1.2) | 1.0(0.9-1.1) | 0.051 |
| Fibrinogen, median(IQR) | 3.2(2.8-3.8) | 3.2(2.4-3.9) | 0.754 | 3.4(2.8-4.3) | 3.1(2.6-3.7) | 0.001 |
| D-dimer, median(IQR) | 0.3(0.2-0.7) | 0.2(0.1-0.7) | 0.321 | 0.4(0.2-0.8) | 0.3(0.2-0.6) | 0.117 |
| HbA1C, median(IQR) | 5.5(5.3-6.3) | 5.8(5.3-6.8) | 0.380 | 5.7(5.4-6.5) | 5.8(5.4-7.0) | 0.495 |
| Prolactin, median(IQR) | 13.4(9.2-24.6) | 15.3(9.8-21.5) | 0.647 | - | - | - |
| Estradiol, median(IQR) | 32.5(7.1-111.8) | 18.1(5.0-59.5) | 0.061 | - | - | - |
| Testosterone, median(IQR) | - | - | - | 3.7(3.0-4.7) | 3.9(3.0-5.2) | 0.231 |
| Interleukin 1β, median(IQR) | 74.8(33.3-172.2) | 82.0(38.2-214.0) | 0.719 | 74.4(34.0-171.2) | 71.2(23.3-171.1) | 0.691 |
| Interleukin 6, median(IQR) | 6.1(2.3-13.1) | 6.0(2.5-9.5) | 0.285 | 6.0(2.5-11.7) | 6.0(3.0-8.7) | 0.247 |
| Interleukin 10, median(IQR) | 11.2(5.7-46.6) | 11.6(1.3-33.3) | 0.244 | 9.3(3.4-26.8) | 9.5(3.9-21.7) | 0.912 |
| Interleukin 18, median(IQR) | 1557.6(445.1-3584.2) | 1614.7(643.0-2690.6) | 0.752 | 2098.4(925.3-5448.2) | 2162.9(1046.6-4914.9) | 0.806 |
| TNF-α, median(IQR) | 39.3(16.8-52.0) | 33.7(14.1-57.7) | 0.888 | 38.6(20.8-60.4) | 40.4(23.2-61.2) | 0.437 |
| BDNF, median(IQR) | 3.4(1.8-6.0) | 7.0(2.7-12.8) | 0.001 | 4.1(2.4-8.2) | 4.0(2.3-8.7) | 0.970 |
| Interferon-γ, median(IQR) | 4.9(1.9-9.0) | 4.9(2.5-10.7) | 0.596 | 5.8(2.4-9.8) | 4.5(1.5-7.8) | 0.012 |
| Fasting C peptide, median(IQR) | 1.7(1.2-2.2) | 1.9(1.3-2.3) | 0.565 | 2.1(1.5-2.6) | 1.9(1.1-2.5) | 0.018 |
| Cortisol, median(IQR) | 11.4(9.1-15.0) | 13.0(10.4-15.5) | 0.429 | 13.0(10.5-16.1) | 12.6(10.2-15.9) | 0.425 |
| ACTH, median(IQR) | 22.2(15.5-35.9) | 22.8(10.3-38.9) | 0.484 | 33.5(18.7-48.5) | 29.6(14.8-46.5) | 0.163 |

BMI:body mass index; CHD:coronary heart disease; HDL:high-density lipoprotein; LDL:low-density lipoprotein; CRP:hypersensitive C-reactive protein;TSH: thyroid stimulating hormone; FT3:free triiodothyronine; FT4:free tetraiodothyronine; HbA1C:glycosylated hemoglobin; TNF-α:tumor necrosis factor-α; BDNF: brain-derived neurotrophic factor; ACTH:brain-derived neurotrophic factor

Table 6. The association between NIHSS score and PSD in female and male

| Variable | Female | | | Male | | |
| --- | --- | --- | --- | --- | --- | --- |
|  | PSD(n=44) | Non-PSD(n=71) | p value | PSD(n=125) | Non-PSD(n=290) | p value |
| NIHSS score, median(IQR) | 1.5(1-3) | 2(1-2) | 0.979 | 1(1-2) | 1(1-2) | 0.434 |

In females, multivariate logistic regression analysis performed using only clinical variables found no significant variables. When both clinical variables and blood biomarkers were included for multivariate logistic regression analysis, significant differences were only found for blood biomarkers (magnesium, FT3 and BDNF) between the PSD and non-PSD groups (Table 7). In males, multivariate logistic regression analysis using only clinical variables revealed exercise habit as the only significantly different clinical variable (Table 8). When both clinical variables and blood biomarkers were included for multivariate logistic regression analysis, significant differences in clinical variables and blood biomarkers (exercise habit, fibrinogen and FT4) were found between the PSD and non-PSD groups (Table 9). Although FT4 was found to be significantly different in multivariate logistic regression in males, the difference was not significant after multiple correction. Therefore, the results of the traditional regression analysis and PLS-DA analysis were consistent.

Table 7. Multivariate analysis of clinical variables and blood biomarkers in PSD and non-PSD of female

| Variables | β | p | OR | 95%CI |
| --- | --- | --- | --- | --- |
| Magnesium | -7.878 | 0.003 | 0.001 | 0.001-0.071 |
| FT3 | -0.770 | 0.004 | 0.463 | 0.273-0.784 |
| BDNF | -0.088 | 0.029 | 0.916 | 0.846-0.991 |

FT3:free triiodothyronine; BDNF: brain-derived neurotrophic factor

Table 8. Multivariate analysis of clinical variables in PSD and non-PSD of male

| Variables | β | p | OR | 95%CI |
| --- | --- | --- | --- | --- |
| Exercise habit | -0.767 | 0.001 | 0.464 | 0.296-0.729 |

Table 9. Multivariate analysis of clinical variables and blood biomarkers in PSD and non-PSD of male

| Variables | β | p | OR | 95%CI |
| --- | --- | --- | --- | --- |
| Exercise habit | -0.772 | 0.001 | 0.462 | 0.292-0.731 |
| Fibrinogen | 0.294 | 0.004 | 1.342 | 1.096-1.643 |
| FT4 | 0.038 | 0.049 | 1.039 | 1.004-1.643 |

FT4:free tetraiodothyronine
